# Recent advances in understanding the neonatal microbiome

**DOI:** 10.12688/f1000research.22355.1

**Published:** 2020-05-22

**Authors:** Matthew J. Dalby, Lindsay J. Hall

**Affiliations:** 1 Gut Microbes and Health, Quadram Institute Bioscience, Norwich Research Park, Norwich, UK; 2 Intestinal Microbiome, School of Life Sciences, Technical University of Munich, Freising, Germany; 3 ZIEL – Institute for Food & Health, Technical University of Munich, Freising, Germany

**Keywords:** neonatal, microbiome, gut, transmission, diet, antibiotics, preterm infants

## Abstract

The neonatal developmental window represents a key time for establishment of the gut microbiota. First contact with these microbes within the infant gastrointestinal tract signifies the start of a critical mutualistic relationship, which is central for short- and longer-term health. Recent research has provided insights into the origin of these microbial pioneers, how they are maintained within the gut environment, and how factors such as antibiotics or preterm birth may disrupt the succession of beneficial microbes.  The acquisition, colonisation, and maintenance of the early life microbiota, and subsequent interactions with the host is a rapidly developing research area. In this review we explore some of these key topics which have been illuminated by recent research, and we highlight some of the important unresolved questions which currently limit our overall understanding of the neonatal gut microbiome.

## Introduction

The communities of microbes that inhabit the infant gut play numerous important roles across the early life developmental window that directly impacts neonatal health. The gut microbiota is involved in the programming and maturation of the immune system ^1^, the use and modification of dietary nutrients, shaping the gut environment by producing metabolites as by-products of their metabolism ^2^, and preventing colonisation of the gut by pathogens. The neonatal period after birth (which for this review we define as the first month after birth) is a crucial phase for the establishment of early life microbial pioneers, which helps establishment of the wider microbial community over time. Here we will focus on the infant gut, which represents the (to date) most studied microbiota site and the body niche harbouring the most diverse and dense microbial community.

**Figure 1.**
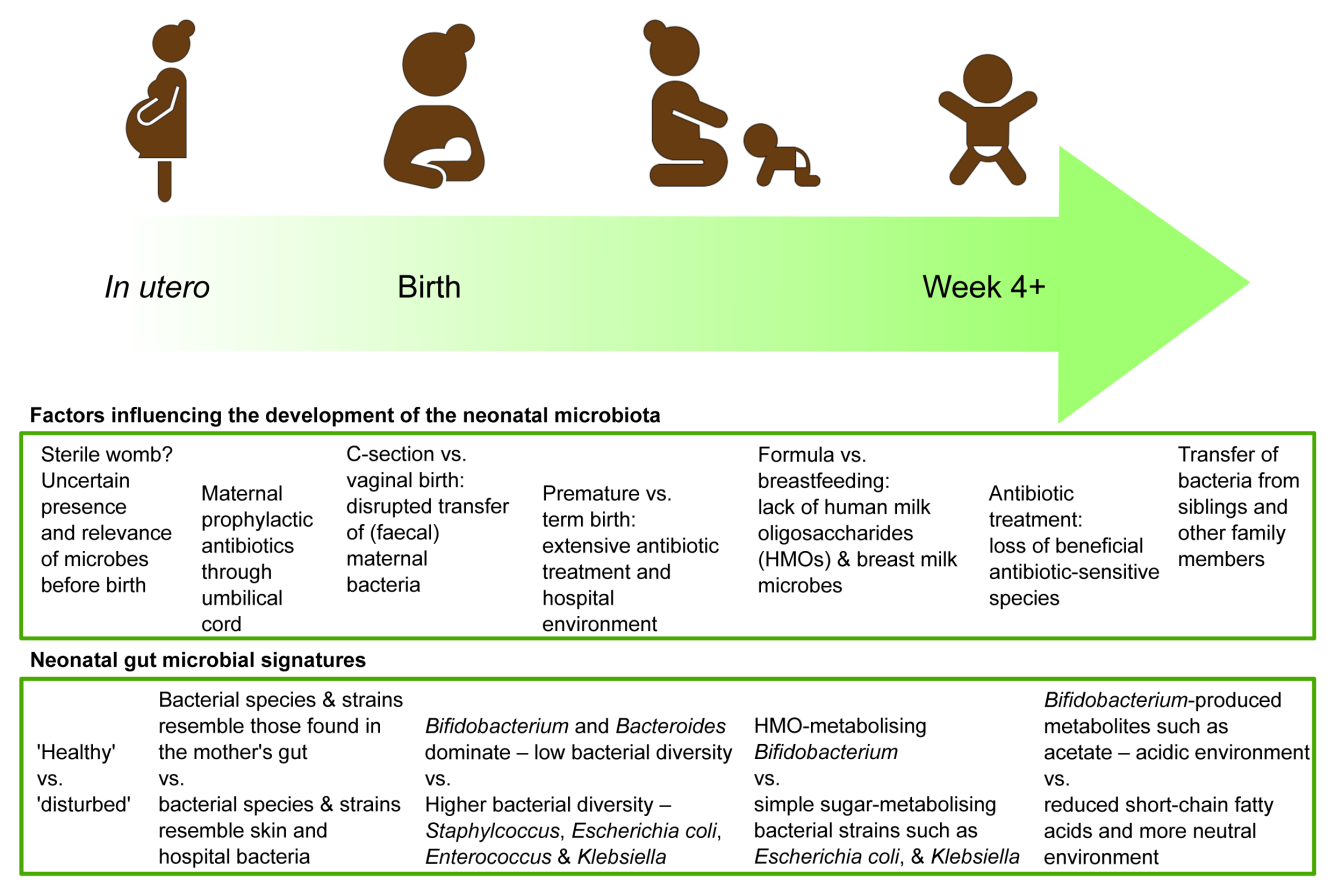
A summary of current understanding of factors influencing the establishment of the neonatal microbiota and the resulting microbial signatures of a “healthy” neonatal microbiota.

Compared to the adult gut, the neonatal infant gut hosts a relatively uncomplicated community of bacteria, fungi, and viruses. Of these, bacteria have been the focus for many researchers, while the presence of fungi ^3^ and viruses ^4^ is only now receiving due attention, and the effects of their presence remain little explored compared to their bacterial neighbours ^5– 8^. From initial colonisation at birth, the infant acquires a community of microbes specialised at inhabiting the human gut, which evolves and changes through infancy and childhood. These changes occur primarily in response to a changing nutritional environment, with other external factors such as antibiotics also significantly impacting community composition ( Figure 1).

The bacterial genus *Bifidobacterium* is a “characteristic” member of the infant gut and typically dominates the microbiota in vaginally delivered, breastfed infants ^9^. Specific species and strains of *Bifidobacterium* have evolved to selectively digest special sugars in breast milk. *Bifidobacterium* metabolise these sugars, producing various microbial fermentation products such as the short chain fatty acid acetate, which reduces pH, creating an acidic gut environment ^2^, whilst also metabolising breast milk amino acids into aromatic lactic acid, which has emerging roles that include improving the integrity of the infant gut wall ^10^. While the importance of the gut microbiota and its interactions with the infant are now clear, the ways in which an infant acquires their microbiota and the source of these microbes have until recently remained largely unknown.

## Origin of the neonatal microbiota

The initial, and probably most important, contribution to the establishment of the infant microbiota is microbes from the infant’s mother, acquired by vertical transmission ^11^. In the womb, developing infants remain largely isolated from exposure to microorganisms in the environment ^12^. During and shortly after birth, the infant is rapidly exposed to microbes that may colonise transiently or may find a longer-term niche. The methods of delivery play a significant role in determining this initial ‘inoculation’. Studies have shown differences in microbial composition of the infant gut between those born by vaginal delivery and those born by caesarean delivery. Although previous research has been conflicting on its impact, recent larger pregnancy–infant cohort studies have shown that delivery method, either vaginal or caesarean birth, does result in a different gut microbial signature, highlighting the importance of the first microbes to which an infant is exposed ^13– 15^. Infants delivered by caesarean section appear to have disrupted transfer of *Bacteroides* and *Bifidobacterium* from the mother, with increased colonisation by opportunistic pathogens found in the hospital environment such as *Enterococcus*, *Enterobacter*, and *Klebsiella* species ^13^. The “disturbed” microbiota associated with caesarean section has prompted some to attempt “vaginal seeding” ^16^; this is the deliberate transfer of the vaginal microbiota to the newborn infant to promote the establishment of a “normal” infant microbiota. However, this practice has recently been called into question, as the vaginal microbiota is not similar to the microbiota that typically soon comes to dominate the infant gut, alongside the risk of group B streptococcus (GBS) transfer (see antibiotic section below). Indeed, a recent review indicated that the differences seen in caesarean-born infants may be due to factors beyond a lack of exposure to vaginal microbes (e.g. antibiotic usage), and other studies suggest the maternal gut microbiota (and cross-contamination and transfer during childbirth) may also play a key role in the establishment of these first microbes ^13, 16^.

Alongside transfer of members of the maternal vaginal and gut microbiota, other body sites such as the skin also harbour microbes that are typical members of the very early life infant gut, e.g. *Streptococcus* and *Staphylococcus*. However, after the first week, bacteria specialised in inhabiting the gut rapidly start to dominate ^17^. The implications of this initial transient colonisation with bacteria originating from the vagina, mouth, and skin currently remain unclear but may link to establishment of an anaerobic environment by these typically facultative anaerobes (that use up the oxygen in the neonatal gut), which in turn facilitates colonisation by other more specialised (anaerobic) microbiota members. There is also evidence that strains of bacteria acquired from mothers are more likely to adapt to and persist in the infant gut than bacteria colonising from other sources ^17^.

During early infancy, other close family members may also act as sources of bacterial colonisation of the infant gut, with these microbes acquired by horizontal transmission. A recent example of this from Japan has shown that a traditional Japanese custom of sharing bathtub water was linked to the transfer of *Bifidobacterium longum* between family members ^18^. Interestingly, some common species of adult gut bacteria, including members of Clostridia and *Akkermansia muciniphila*, appear to be absent, or only present in low levels in the first year of life, and once established do not appear to originate from the mother ^19^. Acquisition of these new microbes may be enabled by the ability among many microbiota members (including Clostridia) to form protective endospores, allowing them to survive outside the gut for prolonged periods of time ^20^.

Breast milk has been found to contain microbes and has recently emerged as another source of microbes for the infant gut. Suggested origins for the bacteria present in breast milk include external transfer into the milk ducts during feeding and internal transfer from the maternal gut to the breast. Many of the bacteria detected in milk samples were not found in the infant gut and *Bifidobacterium*, the most abundant bacteria in the infant gut, was found in only 40% of breast milk samples, suggesting that breast milk may act as an additional source of colonisation ^21^. The importance of the milk microbiota remains to be explored, including the origin of these bacteria and other microbial groups, such as fungi, the presence of which has been recently reported in breast milk samples ^22^.

The womb has traditionally been considered largely sterile; however, some previous studies detected microbial signatures after DNA sequencing of placenta, amniotic fluid, and meconium samples ^23^. The inherent problems associated with sequencing low biomass samples like these that contain very low quantities of DNA are a matter of ongoing debate, with recent comprehensive carefully controlled studies indicating that all aspects of sample collection and preparation and downstream sequencing likely introduce contaminants observed in previous studies ^24^. If indeed present, the low DNA yields indicate that any bacteria in the womb would be in very low numbers, with the various genus of bacteria identified not appearing to colonise the infant after birth. Thus, the effect of such bacterial exposure before birth is unlikely to form a key pathway for seeding of the neonatal gut microbiota.

Whilst these studies have shed some light on initial colonisation of the infant gut and microbial succession dynamics, there remains much to be uncovered as to the various routes of microbes into the infant gut. While research so far has focused on bacteria, there is an emerging world of viruses and fungi whose origin, transmission, and establishment in the gut remain unknown, including how these communities of microbes interact with each other during these very first ecological stages.

## Shaping the microbiota

### Human milk oligosaccharides

After colonising the infant gut, the composition of this new microbiota is shaped by diet and the components of that diet available to feed those bacteria present, i.e. breast milk or formula (or both). Breast milk is a complex biological fluid with many different nutritional and host components, such as enzymes and antibodies, and exclusive breastfeeding for up to 6 months is supported by WHO and UNICEF as the gold standard for infant nutrition ^25^. Human milk oligosaccharides (HMOs) are chains of sugars found in human breast milk, and over 200 different types have been identified so far. They are not broken down by digestive enzymes produced by the infant and pass undigested into the infant’s lower intestine. HMOs have co-evolved to feed and encourage the establishment of beneficial species and strains of *Bifidobacterium* that produce special enzymes to break down these complex sugars. They also signal to the cells lining the infant gut and act as decoys to which pathogenic bacteria attach, hampering their ability to colonise ^26^. Owing to a strong bifidogenic effect, exclusive feeding with breast milk can bring the gut microbiota of caesarean-born infants closer to that of vaginal-born infants by selectively feeding the *Bifidobacterium* present ^27^.

The HMOs in breast milk are synthesised in the mammary gland. Their amount and composition vary between women and over the course of lactation. HMO concentration is higher during the early stages of lactation and decreases gradually over time ^28^. Differences between women are associated with the genetic status of the mother (i.e. linked to Lewis blood type [FUT3] and secretor status [FUC2]), and these differences in mothers’ milk may support different bifidobacterial communities within the infant gut ^29^. This raises questions for future research about whether different *Bifidobacterium* species and strains are better suited to particular maternal milk profiles.

*In vitro* studies have shown that HMOs promote the growth of certain, but not all, *Bifidobacterium*. The breakdown of HMOs is not a simple process of a specific HMO feeding a particular bacterial strain; recent research has shown that cross-feeding takes place within communities of different species and strains of *Bifidobacterium* ^30^. Some strains can start the breakdown of these complex sugars and then others may make use of the by-products to fuel their own metabolism.

As understanding of the importance of HMOs in breast milk has increased, efforts have focused on synthesising individual HMOs, resulting in the production of two of the most common HMOs in milk: 2’fucosyllactose (2’FL) and lacto-N-neotetraose (LNnT). With the aim of adding one or two of these HMOs to infant formula (to bring it closer to human milk) ^31^, the first formula milks containing 2’FL and LNnT have recently been trialled, funded by the formula producer Nestle, and were reported to be safe and have beneficial effects ^32^. Other companies are now also actively moving into this rapidly emerging area of infant nutrition; however, one or two HMOs added to formula are unlikely to fully replicate the effects of the 200+ different HMOs identified so far in breast milk.

### Antibiotic treatment

While the infant diet feeds different bacteria in the infant gut, the treatment of infections with antibiotics shapes the infant microbiota by killing susceptible bacteria ^33^. Before and during birth, maternal treatment with prophylactic antibiotics can also influence bacterial colonisation.

Antibiotic treatment prior to birth (in mothers) appears to alter infant microbiota composition. GBS is an important pathogen that can cause severe bacterial infections in young infants. To prevent transmission, mothers positive for GBS receive a preventative dose of antibiotics, called intrapartum antibiotic prophylaxis, before vaginal delivery to suppress the transfer of GBS to the infant. However, this practice exposes the infant to antibiotics through the umbilical cord and has profound effects on the infant gut intestinal microbiota, diminishing beneficial commensals such as *Bifidobacterium* and increasing potential pathogenic bacteria such as *Escherichia* and *Enterococcus* ^34^. Prophylactic antibiotics are also routinely used in caesarean section births to prevent infections, and this may also contribute to the differences in microbiota seen in caesarean-born infants. However, recent studies (controlling for such variables) indicate that caesarean section birth alone impacts the microbiota and potential subsequent immune programming ^35^ and that reduced *Bifidobacterium* was independent of prophylactic antibiotic exposure ^36^. Such prophylactic antibiotics are necessary to prevent serious illness in infants; however, further work is required to understand the potential short- and longer-term impact on the infant microbiota.

## Preterm infants

Infants born prematurely before 37 weeks of gestation show important differences in the microbial colonisation of their gut due to their immaturely developed gut, antibiotic treatment, and neonatal intensive care hospital environment ^37^. The gut microbiota of premature infants is characterised by potentially pathogenic types of bacteria that are commonly found in the hospital environment and low levels of *Bifidobacterium*. The transmission and establishment of a normal infant microbiota is disrupted by initial prophylactic antibiotic treatment, followed by often regular antibiotic treatments. In extremely premature infants, the gut itself may also be immature and less suitable for colonisation.

The abnormal microbiota common in premature infants and their underdeveloped gut and immune system leave them vulnerable to diseases such as necrotising enterocolitis (NEC) and sepsis, which are often caused by antimicrobial-resistant bacteria ^38, 39^. These rarely affect full-term infants but are serious and potentially fatal illnesses in premature infants. The prevention of NEC has encouraged efforts to “normalise” the premature infant microbiota, which include inoculating the infant gut with beneficial probiotic strains of bacteria and encouraging breastfeeding (supplemented with donor breast milk) ^40^. Several clinical trial reviews indicate that providing probiotic bacteria to premature infants decreases NEC rates ^10, 41, 42^. However, different species and strains of bacteria are available as potential probiotics to supplement infants, and as yet there is a lack of clear evidence or guidance as to which ones are most effective, either individually or in combination, and why some have failed to provide a benefit ^43^. Understanding how to choose the right species and strain that can colonise the infant’s gut and digest the food available (i.e. breast milk) is key to making probiotic treatments more effective in premature infants.

## Consequences of a disrupted early life microbiota

Several factors can disrupt the transfer and establishment of the infant microbiota, resulting in an abnormal microbiota composition, but whether these early microbial differences persist into later childhood is still unclear. However, as the early gut microbiota coincides with the immune priming window, with work indicating certain species and strains train and mature the immune system, early differences may have long-term effects on future health ^44^. The relative abundance of the bacterial genera has been reported to be decreased in the gut of infants at risk of asthma ^45^. A recent review of the evidence found that overall in infants, greater levels of Bacteroidaceae, Clostridiaceae, and Enterobacteriaceae and lower levels of Bifidobacteriaceae and Lactobacillaceae were associated with higher occurrence of allergies, eczema, or asthma ^46^. Although interesting, the observational nature of research linking early differences in infant gut microbiota to later health problems often does not account for potentially important confounding factors ^47^. There may be other positive influences of the infant microbiota on immunity beyond just avoiding allergic problems. Associations between higher *Bifidobacterium* in early infancy and better immune system responses to vaccination, potentially enhancing immunologic memory, have been reported ^48^. Working out which of these relationships are causal and how they can be manipulated to effectively prevent later-life health problems will require much more basic and translational research.

## Future research

Whilst bacteria have now been relatively well studied/profiled in the infant gut, there is much left to do with respect to understanding direct mechanisms governing microbe–microbe and microbe–host crosstalk. The additional microbial “dark matter”—the potentially large sections of the infant gut microbiota comprising fungi, viruses, and eukaryotic organisms—remains to be explored. Their presence, where they come from, and their effects in infants remain unknown. The use of faecal samples to explore infant gut microbiota is a limitation and is not necessarily representative of the sites of microbial colonisation higher up the infant gut, although access to these mucosal sites is often extremely difficult in neonatal patients or almost impossible in healthy infants.

Areas of the world where the study of the infant gut microbiota has taken place may have masked how the microbiota is changing, and greater research and comparisons with infants in low- and middle-income country settings may give a broader picture. While *Lactobacillus* is not generally considered a component of the infant gut after the first week of life, research from India has found both its presence and its beneficial role in preventing sepsis when isolated and supplemented to infants ^49^. Recent comparisons of infants from Indonesian and New Zealand infants showed that the bacterium *Bifidobacterium longum* subsp. *infantis* dominated the microbiota of Indonesian infants, while a different species, *Bifidobacterium longum* subsp. *longum*, dominated in New Zealand infants ^50^. Moreover, variation in microbiota members and their components, e.g. *Bacteroides* and *Escherichia coli* lipopolysaccharide, may also lead to differential immune programming and subsequent risk of autoimmune conditions in childhood and later life ^51^. Therefore, what is considered normal “here” may not be normal in other geographic regions of the world.

## Conclusions

The establishment of the gut microbiota in infants is an ecological succession shaped by sources of exposure to different microbes over time, which can be potentially disrupted by antibiotics, prematurity, delivery, and diet ( Figure 1). The routes of vertical maternal transmission at birth and later acquisition from other sources need to be better understood in order to correct the disruption caused by necessary medical interventions.
